# An analysis of slippage effects on a solid sphere enclosed by a non-concentric cavity filled with a couple stress fluids

**DOI:** 10.1038/s41598-023-46099-8

**Published:** 2023-11-10

**Authors:** Amal Al-Hanaya, Shreen El-Sapa

**Affiliations:** 1 Department of Mathematical Sciences, College of Science, Princess Nourah Bint Abdulrahman University, 11671 Riyadh, Saudi Arabia; 2 Department of Mathematics, Faculty of Science, Damanhour University, Damanhour, Egypt

**Keywords:** Applied mathematics, Computational science

## Abstract

This investigation shows the effect of slippage on the slow spinning of a rigid sphere covered by a non-concentric spherical hollow full of an incompressible couple stress fluid. Moreover, the velocity slip conditions are employed on surfaces of both the rigid sphere and the cavity. In addition, the solid sphere and the cavity are rotating axially at various angular speeds. The solution is obtained semi-analytically at low Reynolds numbers utilizing the superposition with the numerical collocation approach. This paper discusses the hydrodynamic couple exerted by the fluid on the internal particle. The dimensionless torque increases as the slip and spin slip increase by 99%, the couple stress parameter by 49%, and the separation parameter by 79%. Additionally, the non-dimensional torque decreases with the increase of the size ratio by 89%. Consequently, it is found that all the results agreed with the corresponding numerical analysis in the traditional viscous liquids and the revolving of two eccentric rigid spheres with no slippage (Al-Hanaya et al. in J. Appl Mech Tech Phys 63(5):1–9, 2022).

## Introduction

Recent research on the theory of couple stress fluid behavior is very helpful, and these analyses explain enough about the habits of rheological compound liquids like suspensions of polymers with molecules from prolonged chains, lubricants, liquid crystals, and blood. Therefore, the theory of couple stress fluid, which Stokes first proposed in 1966^1^, is the sole theory of many polar fluids that also be composed of couple stresses and contain the typical Cauchy stress. Couple stress fluids are fluids that are suspended in a viscous medium with stiff particles that are randomly oriented. This theory, which is the most direct popularization of the theory of the traditional fluid, was created by Stokes. Moreover, a couple of conflicts and physical ties are maintained. The theory of couple stress fluid is thoroughly outlined in the work by Stokes^2^. Numerous studies into a few stress fluids, such as^3–5^. Recently, Al-Hanaya et al.^6^ examined the axisymmetric motion of fluid couple stress between two eccentric spinning spheres that the normalized torque on the solid sphere rotates inside the outside sphere with no slippages. Moreover, the normalized torque increases with both the size ratio and separation distance. The couple stress fluids have been applied in several applications of porous mediums such as El-Sapa and Almoneef^7^ investigated the axisymmetric movement of an aerosol particle contained in a couple-stress fluid moving in a slippage regime. On the other hand, Maurya et al.^8^ evaluated the couple stress fluid flow surrounding a solid sphere in a porous material in the presence of a homogeneous magnetic field.

In the study of fluid dynamics, employing the traditional no-slip boundaries condition is prevalent. Consequently, the no-slip requirement, however, may not always hold, and fluid particle slippage on the surface of the stiff barrier can occur, according to various investigations conducted in the past century^9,10^. Additionally, Ellahi^11^ examined how the slip boundary condition affected non-Newtonian flows in a channel. He used the symmetric analysis method (HAM) to solve nonlinear boundary value governing problems. The flows of Couette, Poiseuille, and modified Couette of an incompressible even-pressure flow between parallel walls were all correctly solved by Devakar et al.^12^ using slip limit conditions. The solution is like a viscous classical Newtonian fluid solution with pair pressures close to zero in the finite state. Additionally, the results demonstrate that different pressures reduce fluid velocity. A rigid sliding ball's creeping motion in an infinite binary stress fluid was studied by Ashmawy^13^. He also added the vanishing couple stress condition and the linear sliding limit condition to the sphere's surface. Thus, Slippage conditions at the boundary are used to study the unstable flow of an incompressible couple stress fluid between two plates that are in parallel. by Saad and Ashmawy^14^. Further, the two rigid spheres with slippage surfaces that have distinct diameters and are suspended in a porous medium in a horizontal magnetic field were studied semi-analytically for the Stokes flow approximation^15^.

Medical and industrial applications require porous material fluid flow quantification. Chemical, biological, and environmental engineering and research gently spin a solid particle in an incompressible porous medium, non-Newtonian, or Newtonian fluids. Furthermore, Jeffrey solved limitless Newtonian fluid spheroid rotation^16^. The Stokes flow spinning of a spheroid around its axis of revolutions in a viscous fluid with the slipping influence was further studied by Chang and Keh^17^ where the torque monotonically decreases with the slippage parameter for a spheroid with a constant size ratio. On the other side, at low Reynolds numbers, Lee and Keh studied the static rotation of a sliding spherical particle about the longitudinal axis of a sliding circular tube^18^. The creeping flow of continuous spinning of a slipping sphere that is only slightly distorted in a Brinkman medium was investigated analytically in^19^.

In actual instances of rotation of particles, solid walls surround the fluid in its immediate vicinity^20^. Therefore, it is important to understand if particle rotation is impacted by the presence of boundary walls. So, the slow rotations of a hard sphere near one or more planar walls^21^, inside a spherical hole^22^ were studied. Also, theoretical research has investigated the rotary motion of a soft or porous circular object with a low Reynolds number in a cylinder^23^ and a spherical cavity^24,25^. These investigations demonstrate that borders can have a significant and fascinating impact on how particles rotate. In addition, numerous researchers used the collocation method to solve various fluid dynamics problems. El-Sapa^26^ studied the axisymmetric motion of two rigid spheres in a Brinkman medium with slip surfaces. Sherief et al.^27,28^ discussed the problems of low Reynolds number micropolar fluid motion. The boundary collocation method is widely employed to resolve flow problems^29–32^.

The purpose of this study is to provide semi-analytical solutions for the axisymmetric rotation of a solid sphere within a non-concentric hollow filled by a couple stress fluids under the impact of slippage surfaces. As a result, the boundary collocation procedure is employed in the system of equations. Additionally, given various values of the relevant parameters such as the slippage, the separation, the size ratio, the couple stress, and the angular velocity ratios, the normalized torque acting on the interior solid sphere can be calculated. In general, the results showed good convergence across the parameters considered, and all the results are displayed graphically and calculated tabularly. Several studies have investigated this problem in viscous fluids and microfluidics, but this work concentrates on two concentric spheres with slippages in couple stress fluids.

## Mathematical formulation

By the notion of a low Reynolds number, without the presence of body forces and body couples, the field constraints controlling the steady motion of an incompressible couple stress liquid are dictated by^2^:

1 $$ \nabla \cdot \vec{u} = 0, $$

2 $$ \nabla p + \mu \nabla \wedge \,\nabla \wedge \vec{u} + \eta \,\nabla \wedge \nabla \wedge \nabla \wedge \nabla \wedge \vec{u} = 0. $$

Here, the constant $\mu \,$ is the fluid viscosity, $\eta$ is the viscosity of 1st couple stress, $\vec{u}$ is the velocity vector of the fluid, and $p$ is the fluid pressure. If the relation (2) tends to the classical equation of Navier–Stokes. The tensor forms of $t_{ij}$ and $m_{ij}$ are^2^

3 $$ t_{ij} = - p\,\delta_{ij} + \,2\mu \,d_{ij} - \tfrac{1}{2}\,\,e_{ijk} \,m_{sk,s} , $$

4 $$ m_{ij} = m\delta_{ij} + 4(\eta \,\omega_{j,i} + \eta^{\prime}\,\omega_{i,j} ), $$

where $\eta^{\prime}$ is 2nd couple stress, $m$ is a tensor trace of couple stress, the two tenors are Kronecker delta, $e_{ijk}$ is alternating tensor, $d_{ij}$ is deformation rate tensor, and $\,\vec{\omega }$ is vorticity vector, the two last concepts are formed as:

5 $$ \,d_{ij} = \frac{1}{2}(u_{i,j} + u_{j,i} )\,\,,\,\,\,\,\,\,\omega_{i} = \,\frac{1}{2}e_{ijk} \,u_{k,j} . $$

The enforced boundary constraints may be used to derive the scalar quantity that was mentioned in relation (4). Additionally, one may explicitly define it as^1^ by using the second relation of (4) and the concept of (5):

6 $$ m = \,\frac{1}{3}m_{ii} . $$

The physical constants in the fundamental Eqs. (3) and (4), as well as the equation for movement (2), are presumed to adhere to the following constraints in^2^:

7 $$ \mu \ge 0,\,\,\eta \ge 0,\,\,\eta \ge \eta^{\prime}. $$

Furthermore, suppose that the surface of the sphere is subject to the following circumstances., $r = a$:

- Slippage restriction
  8 $$ \beta \,\left( {u_{\phi } - a\,\Omega \,\sin \theta } \right) = t_{r\phi } , $$
  where is the slippage parameter changing its values from zero to infinity. This coefficient is only related to the type of fluid and the material’s surface. Furthermore, the perfect slip situation becomes possible when the slip coefficient disappears, and the traditional no-slip case may be inferred as a specific instance in this study when the slip parameter approaches infinity. The slippage condition of the boundary has recently been used to solve several viscous fluids^9–12^ and micropolar fluids^26,27^ issues.
- The prevailing condition is the absence of couple stresses^2^
  9 $$ m_{ij} \,n_{i} = 0\,\,\,\,\,\,\,\,\,\,\,\,{\text{on}}\,\,\,\,\,\,\,r = a, $$
  where is the unit normal to the surface of the solid sphere. Stokes^1^ has suggested the boundary conditioning Eq. (9). Only in this situation can mechanical interactions at the borders produce a force distribution., according to physical theory.

## Solution of the problem

Assume that the rotational movement of a spherical object of radius moves symmetry about its axis within an incompressible couple stress liquid. The spherical systemic procedure is established at the center of the sphere, the field functions are not dependent on $\phi$. Further, the velocity and vorticity vectors are represented by:

10 $$ \overrightarrow{u} = \left( {0,\,\,\,0,\,\,\,u_{\phi } (r,\theta )} \right),\, $$

11 $$ \,\overrightarrow{\omega } = \left( {\omega_{r} (r,\theta ),\,\,\omega_{\theta } (r,\theta ),\,\,0} \right), $$

Substitute Eq. (10) into the momentum Eq. (2) by eliminating the pressure, the subsequent p.d.e is obtained as:

12 $$ E^{2} \left( {\,E^{2} - \kappa^{2} } \right)\left( {r\sin \theta \,u_{\phi } } \right)\,\,\, = 0,\,\,\,\,\,\,\,\,\,\,\, $$

where the material constant $\,{1 /\kappa } = \sqrt {{\eta/{a_{{}}^{2} \mu }}}$, is taken into consideration as a polarity indicator for the couple stress fluids approach, and the Stokesian indicator of axial motion is:

13 $$ E^{2} = \frac{{\partial^{2} }}{{\partial r^{2} }} + \frac{{1 - \zeta^{2} }}{{r^{2} }}\frac{{\partial^{2} }}{{\partial \zeta^{2} }},\,\,\,\,\,\,\,\zeta = \cos \theta \,. $$

Moreover, from Eq. (5) the non-vanishing vorticity components $\omega_{r}$ and $\omega_{\theta }$, are:

14 $$ \omega_{r} = \frac{1}{2}(\nabla \times \overrightarrow{u}) \cdot \overrightarrow{e}_{r} = \frac{1}{2}\frac{1}{r\,\sin \theta }\frac{\partial }{\partial \theta }\left( {\,\sin \theta \,u_{\phi } } \right). $$

15 $$ \omega_{\theta } = \frac{1}{2}(\nabla \times \overrightarrow{u}) \cdot \overrightarrow {e}_{\theta } = - \frac{1}{2}\frac{1}{r\,}\frac{\partial }{\partial r}\left( {\,r\,\,u_{\phi } } \right). $$

Furthermore, the tangential stress is calculated by El-Sapa and Almoneef^7^ as:

16 $$ t_{r\phi } = \mu \,r\frac{\partial }{\partial r}\left( {\frac{{u_{\phi } }}{r}} \right)\user2{ + }\frac{1}{2}\left( {\frac{{\partial m_{r\theta } }}{\partial r} + \frac{1}{r}\left( {2m_{r\theta } + m_{\theta r} } \right) + \frac{\cot \theta }{r}\left( {m_{\theta \theta } - m_{\phi \phi } } \right) + \frac{1}{r}\frac{{\partial m_{\theta \theta } }}{\partial \theta }} \right). $$

We obtain the following couple stresses by using the tensor relation (4):

17a $$ m_{rr} = m + 4\left( {\eta + \eta^{\prime}} \right)\frac{{\partial \omega_{r} }}{\partial r}, $$

17b $$ m_{r\theta } = 4\eta \frac{{\partial \omega_{\theta } }}{\partial r} + 4\eta^{\prime}\frac{1}{r}\left( {\frac{{\partial \omega_{r} }}{\partial \theta } - \omega_{\theta } } \right)\,\,, $$

17c $$ m_{\theta r} = 4\eta \frac{1}{r}\left( {\frac{{\partial \omega_{r} }}{\partial \theta } - \omega_{\theta } } \right) + 4\eta^{\prime}\frac{{\partial \omega_{\theta } }}{\partial r}, $$

17d $$ m_{\theta \theta } = m + 4(\eta + \eta^{\prime})\frac{1}{r}\left( {\frac{{\partial \omega_{\theta } }}{\partial \theta } + \omega_{r} } \right), $$

17e $$ m_{\phi \phi } = m + 4\left( {\eta + \eta^{\prime}} \right)\frac{1}{r}\left( {\omega_{r} + \cot \theta \omega_{\theta } } \right). $$

The differential Eq. (12) has the following generalized solution:

18 $$ u_{\phi } \left( {r,\theta } \right) = \sum\limits_{n = 1}^{\infty } {\left[ {A_{n} \,\,r^{ - n - 1} + B_{n} \,r^{n} + \,C_{n} r^{{\frac{ - 1}{2}}} \,I_{{n + \tfrac{1}{2}}} \left( {\kappa \,r} \right) + D_{n} \,\,r^{{\frac{ - 1}{2}}} K_{{n + \tfrac{1}{2}}} \left( {\kappa \,r} \right)} \right]} \,\,P_{n}^{1} \left( \zeta \right), $$

where the two functions and $K_{n} (.)$ the first and second types of modified Bessel functions of order $n$, respectively. Also, $P_{n}^{1} (.)$ denotes the corresponding Legendre polynomials of order the first type.

Applying Eq. (18) to Eqs. (14) and (15), the vorticity components are obtained as:

19 $$ \omega_{r} = \tfrac{1}{2}\sum\limits_{n = 1}^{\infty } {n\left( {n + 1} \right)\left[ {A_{n} \,\,r^{ - n - 2} + B_{n} \,r^{n - 1} + C_{n} \,\,r^{{\tfrac{ - 3}{2}}} I_{{n + \tfrac{1}{2}}} (\kappa \,r) + D_{n} \,\,r^{{\tfrac{ - 3}{2}}} K_{{n + \tfrac{1}{2}}} (\kappa r)} \right]} \,\,P_{n} \left( \zeta \right), $$

20 $$ \begin{aligned} \omega_{\theta } & = \tfrac{1}{2}\sum\limits_{n = 1}^{\infty } {\left[ {nA_{n} \,\,r^{ - n - 2} - (n + 1)B_{n} \,r^{n - 1} + C_{n} \,\,r^{{\tfrac{ - 3}{2}}} \left( {nI_{{n + \tfrac{1}{2}}} \left( {\kappa r} \right) - \kappa rI_{{n - \tfrac{1}{2}}} \left( {\kappa r} \right)} \right)} \right.} \hfill \\ & \quad \left.  { + D_{n} \,\,r^{{\tfrac{ - 3}{2}}} \left( {nK_{{n + \tfrac{1}{2}}} \left( {\kappa r} \right) + \kappa rK_{{n - \tfrac{1}{2}}} \left( {\kappa r} \right)} \right)} \right]\,P_{n}^{1} \left( \zeta \right). \hfill \\ \end{aligned} $$

The couple is determined by applying Eqs. (19)–(20) to Eq. (17):

21 $$ \begin{aligned} m_{rr} & = m + 2\left( {\eta + \eta^{\prime}} \right)\sum\limits_{n = 1}^{\infty } {n\left( {n + 1} \right)\left[ { - (n + 2)A_{n} \,\,r^{ - n - 3} + (n - 1)B_{n} \,r^{n - 2} } \right.} \, \hfill \\ & \quad \,\,\,\,\,\,\,\left. { + C_{n} \,\,r^{{\tfrac{ - 5}{2}}} \left( {\kappa \,r\,I_{{n - \tfrac{1}{2}}} (\kappa \,r) - (n + 2)I_{{n + \tfrac{1}{2}}} (\kappa \,r)} \right) + D_{n} \,\,r^{{\tfrac{ - 5}{2}}} \left( {\kappa \,r\,K_{{n - \tfrac{1}{2}}} (\kappa \,r) + (n + 2)K_{{n + \tfrac{1}{2}}} (\kappa \,r)} \right)} \right]\,P_{n} \left( \zeta \right), \hfill \\ \end{aligned} $$

22 $$ \begin{aligned} m_{r\theta } & = 2\sum\limits_{n = 1}^{\infty } {\left[ { - n(n + 2)(\eta + \eta^{\prime})\,r^{ - n - 3} A_{n} } \right.} - (n^{2} - 1)(\eta + \eta^{\prime})r^{n - 2} B_{n} \hfill \\ & \quad + r^{{\tfrac{ - 5}{2}}} \left( {\left( {\eta + \eta^{\prime}} \right)\kappa rI_{{n - \tfrac{1}{2}}} (\kappa r) - \left( {\eta (\kappa^{2} r^{2} + n^{2} + 2n) + \eta^{\prime}n(n + 2)} \right)I_{{n + \tfrac{1}{2}}} (\kappa r)} \right)C_{n} \hfill \\  & \quad - r^{{\tfrac{ - 5}{2}}} \left. {\left( {\left( {\eta + \eta^{\prime}} \right)\kappa rK_{{n - \tfrac{1}{2}}} (\kappa r) + \left( {\eta (\kappa^{2} r^{2} + n^{2} + 2n) + \eta^{\prime}n(n + 2)} \right)K_{{n + \tfrac{1}{2}}} (\kappa r)} \right)D_{n} } \right]P_{n}^{1} (\zeta ) \hfill \\ \end{aligned} $$

23 $$ \begin{aligned} m_{\theta r} & = 2\sum\limits_{n = 1}^{\infty } {\left[ { - n(n + 2)(\eta + \eta^{\prime})\,r^{ - n - 3} A_{n} - } \right.} (n^{2} - 1)(\eta + \eta^{\prime})r^{n - 2} B_{n} \hfill \\ & \quad  + r^{{\tfrac{ - 5}{2}}} \left( {\kappa r(\eta + \eta^{\prime})I_{{n - \tfrac{1}{2}}} (\kappa r) - \left( {\eta^{\prime}(\kappa^{2} r^{2} + n^{2} + 2n) + \eta (n + 2)} \right)I_{{n + \tfrac{1}{2}}} (\kappa r)} \right)C_{n} \hfill \\ & \quad \left. {\, - r^{{\tfrac{ - 5}{2}}} \left( {\kappa r(\eta + \eta^{\prime})K_{{n - \tfrac{1}{2}}} (\kappa r) + \left( {\eta^{\prime}(\kappa^{2} r^{2} + n^{2} + 2n) + \eta (n + 2)} \right)K_{{n + \tfrac{1}{2}}} (\kappa r)} \right)D_{n} } \right]P_{n}^{1} (\zeta ) \hfill \\ \end{aligned} $$

24 $$ \begin{aligned} m_{\theta \theta } & = m + 2(\eta + \eta^{\prime})\sum\limits_{n = 1}^{\infty } {\left[ {n\left( {(n + 1)^{2} P_{n} \left( \zeta \right) - \cot \theta P_{n}^{1} \left( \zeta \right)} \right)A_{n} \,\,r^{ - n - 3} } \right.} \hfill \\ & \quad + (n + 1)\left( { - n^{2} P_{n} \left( \zeta \right) + \cot \theta P_{n}^{1} \left( \zeta \right)} \right)B_{n} \,r^{n - 2} + r^{{\frac{ - 5}{2}}} \left( {n(n + 1)\left( {n(n + 2)I_{{n + \tfrac{1}{2}}} \left( {\kappa r} \right) - \kappa rI_{{n - \tfrac{1}{2}}} \left( {\kappa r} \right)} \right)P_{n} \left( \zeta \right)} \right. \hfill \\ & \quad \left. { - \cot \theta \left( {nI_{{n + \tfrac{1}{2}}} \left( {\kappa r} \right) - \kappa rI_{{n - \tfrac{1}{2}}} \left( {\kappa r} \right)} \right)P_{n}^{1} \left( \zeta \right)} \right)C_{n} \, + r^{{\frac{ - 5}{2}}} \left( {n(n + 1)\left( {n(n + 2)K_{{n + \tfrac{1}{2}}} \left( {\kappa r} \right) + \kappa rK_{{n - \tfrac{1}{2}}} \left( {\kappa r} \right)} \right)P_{n} \left( \zeta \right)} \right. \hfill \\ & \quad \left. {\left. { - \cot \theta \left( {nK_{{n + \tfrac{1}{2}}} \left( {\kappa r} \right) + \kappa rK_{{n - \tfrac{1}{2}}} \left( {\kappa r} \right)} \right)P_{n}^{1} \left( \zeta \right)} \right)D_{n} \,\,} \right]\, \hfill \\ \end{aligned} $$

25 $$ \begin{aligned} m_{\phi \phi } & = m + 4\left( {\eta + \eta^{\prime}} \right)\sum\limits_{n = 1}^{\infty } {\left[ {\left( {n\left( {n + 1} \right)P_{n} \left( \zeta \right) + n\cot \theta P_{n}^{1} \left( \zeta \right)} \right)A_{n} \,\,r^{ - n - 3} } \right.} \hfill \\ & \quad  + \left( {n\left( {n + 1} \right)P_{n} \left( \zeta \right) - (n + 1)\cot \theta P_{n}^{1} \left( \zeta \right)} \right)B_{n} \,r^{n - 2} \hfill \\ & \quad + \,r^{{\frac{ - 5}{2}}} \left( {n\left( {n + 1} \right)I_{{n + \frac{1}{2}}} (\kappa \,r)P_{n} \left( \zeta \right) + \left( {nI_{{n + \frac{1}{2}}} \left( {\kappa r} \right) - \kappa rI_{{n - \frac{1}{2}}} \left( {\kappa r} \right)} \right)P_{n}^{1} \left( \zeta \right)} \right)C_{n} \,\,\, \hfill \\ & \quad  \left. { + \,r^{{\frac{ - 5}{2}}} \left( {n\left( {n + 1} \right)K_{{n + \frac{1}{2}}} (\kappa r)P_{n} \left( \zeta \right) + \left( {nK_{{n + \frac{1}{2}}} \left( {\kappa r} \right) + \kappa rK_{{n - \frac{1}{2}}} \left( {\kappa r} \right)} \right)P_{n}^{1} \left( \zeta \right)} \right)D_{n} \,} \right]. \hfill \\ \end{aligned} $$

The boundary conditions (9) can be written as

26 $$ m_{rr} \, = 0\,\,\,\,\,\,\,\,\,\,\,\,{\text{on}}\,\,\,\,\,\,\,r = a $$

From Eq. (22)

27 $$ \begin{aligned} m & = - 2\left( {\eta + \eta^{\prime}} \right)\sum\limits_{n = 1}^{\infty } {n\left( {n + 1} \right)\left[ { - (n + 2)A_{n} a_{{}}^{ - n - 3} + (n - 1)B_{n} \,a_{{}}^{n - 2} } \right.} \, \hfill \\ & \quad \left. { + C_{n} a_{{}}^{{\tfrac{ - 5}{2}}} \left( {\kappa \,a\,I_{{n - \tfrac{1}{2}}} (\kappa \,a) - (n + 2)I_{{n + \tfrac{1}{2}}} (\kappa \,a)} \right) + D_{n} a_{{}}^{{\tfrac{ - 5}{2}}} \left( {\kappa \,a\,K_{{n - \tfrac{1}{2}}} (\kappa \,a) + (n + 2)K_{{n + \tfrac{1}{2}}} (\kappa \,a)} \right)} \right]\,P_{n} \left( \zeta \right). \hfill \\ \end{aligned} $$

Employing the obtained Eqs. (18), (21)–(25), and (27) into (16), we get:

28 $$ \begin{aligned} T_{r\phi } & = \,\sum\limits_{n = 1}^{\infty } {\left[ {A_{n} r_{{}}^{ - n - 4} \left( { - n\,(n + 1)(2\eta - n\eta^{\prime}) - \mu r_{{}}^{2} (n + 2) - \alpha_{1r} } \right)} \right.} \hfill \\ & \quad + B\,r_{{}}^{n - 3} \left( { - n(n + 1)(2\eta + (n + 1)\eta^{\prime}) + \mu r_{{}}^{2} (n - 1) + \alpha_{2r} } \right) \hfill \\ & \quad - D_{n} r_{{}}^{{\tfrac{ - 7}{2}}} \left( {\kappa r\left( {\mu r_{{}}^{2} - n\left( {\eta + \eta^{\prime}} \right)\left( {n + 1} \right)\kappa \,r - (\kappa^{2} r_{{}}^{2} + n)\eta - n(n + 1)\eta^{\prime} + \alpha_{3r} } \right)K_{{n - \tfrac{1}{2}}} (\kappa r)} \right. \hfill \\ & \quad \left. { + \left( {\mu \kappa_{{}}^{2} (n + 2) - n(\kappa^{2} r_{{}}^{2} + 3n)\eta + n(n + 1)(2\eta - n\eta^{\prime}) - \alpha_{4r} } \right)K_{{n + \tfrac{1}{2}}} (\kappa r)} \right) \hfill \\ & \quad + C_{n} \,r_{{}}^{{\tfrac{ - 7}{2}}} \left( {\kappa r_{{}} \left( {\mu r_{{}}^{2} + \left( {\eta + \eta^{\prime}} \right)n\left( {n + 1} \right)\kappa \,r - (\kappa^{2} r_{{}}^{2} + n)\eta - n(n + 1)\kappa r\,\eta^{\prime} + \alpha_{5r} } \right)I_{{n - \tfrac{1}{2}}} (\kappa r)} \right. \hfill \\ & \quad \left. {\,\left. { + \left( { - \mu r_{{}}^{2} (n + 2) + n(\kappa^{2} r_{{}}^{2} + 3n)\eta - n(n + 1)(2\eta - n\eta^{\prime}) + \alpha_{6r} } \right)I_{{n + \tfrac{1}{2}}} (\kappa r)} \right)P_{n}^{1} (\zeta )} \right], \hfill \\ \end{aligned} $$

where

$$ \begin{aligned} \alpha_{0r} &= (n^{2} \eta - 2\eta^{\prime})\cot^{2} \theta + ((1 - 2n^{2} )\eta + (3 - n^{2} )\eta^{\prime})\csc^{2} \theta \hfill \\ \alpha_{1r} &= n(n + 1)(n + 2)(\eta + \eta^{\prime}) - 3n^{2} \eta + n\alpha_{0r} \hfill \\ \alpha_{2r} & = (n + 1)\left[ {n(n - 1)(\eta + \eta^{\prime}) + ((n^{2} - n - 2)\eta - 2\eta^{\prime})\cot^{2} \theta - ((2n^{2} - n + 3)\eta + (n^{2} - 3)\eta^{\prime})\csc^{2} \theta } \right] \hfill \\ \alpha_{3r} & = ((n^{2} - 2)\eta - 2\eta^{\prime})\cot^{2} \theta + ((3 - 2n^{2} )\eta + (3 - n^{2} )\eta^{\prime})\csc^{2} \theta \hfill \\ \alpha_{4r} & = n(n + 1)(n + 2)(\eta + \eta^{\prime}) - n\alpha_{0} \hfill \\ \alpha_{5r} & = - (n + 2) + \alpha_{2r} ,\,\,\,\,\, \hfill \\ \alpha_{6r} & = 3n^{2} \eta - \alpha_{1r} . \hfill \\ \end{aligned} $$

As a result of the axisymmetric particle being impacted by the fluid flow, a torque is generated that has a magnitude of^28^

29 $$ T = 8\pi \mu \,\,\mathop {\lim }\limits_{r \to \infty } \frac{{r^{2} q_{\phi } }}{{\sqrt {1 - \zeta^{2} } }}. $$

## Hydrodynamic interaction of a rigid sphere enveloped by a spherical cavity filled with a couple stress fluid

**Figure 1:**
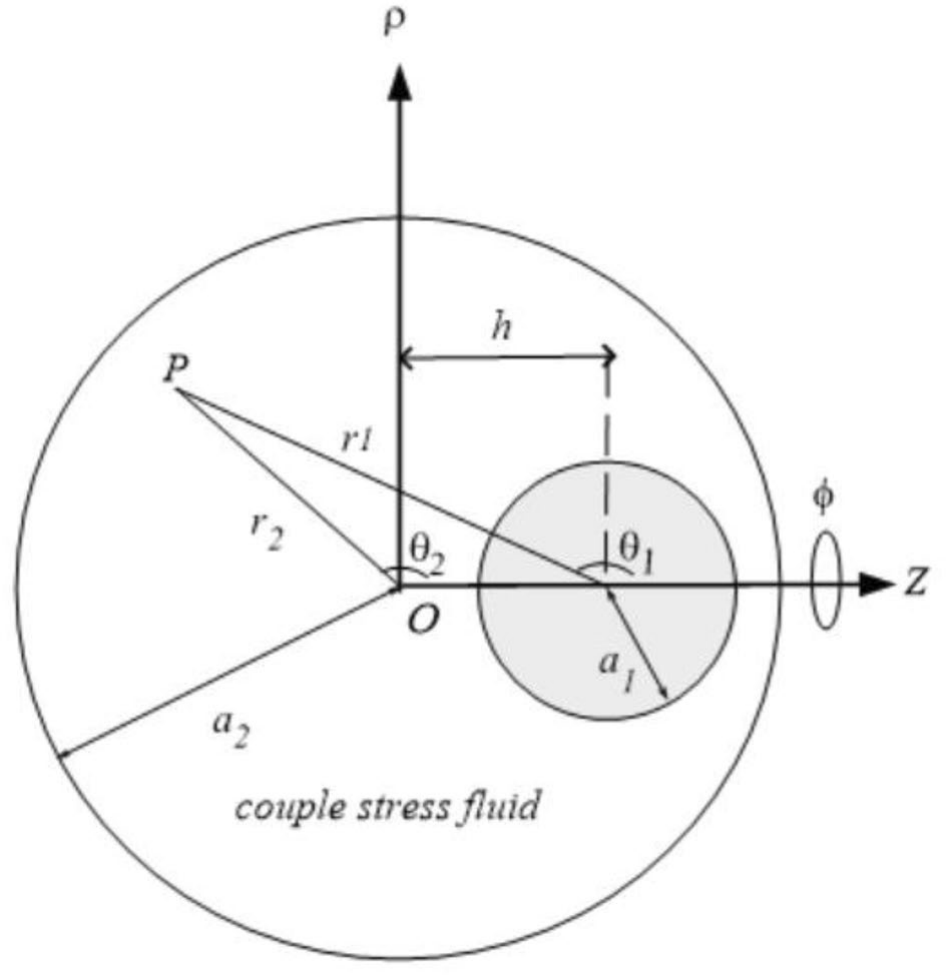
The geometrical shape of a sphere covered by a cavity filled with couple stress fluid.

For this simulation, it is assumed the annulus between the solid sphere, $a_{1}$ and a spherical cavity, $a_{2}$ is filled with a constant density of couple stress liquid. Therefore, the sphere and the spherical cavity are rotating around a connecting line of its centers with distinct angular speeds $\Omega_{1}$ ad $\Omega_{2}$, respectively and at a distance $h$ from their centers as shown in Fig. 1. Consider that $\,u_{\phi }^{(1)} ,\omega_{r}^{(1)} ,\,\,\omega_{\theta }^{(1)}$ are the components of velocity and vorticity as a result of the presence of the solid particle $a_{1}$ without the spherical cavity $a_{2}$ and $\,u_{\phi }^{(2)} ,\omega_{r}^{(2)} ,\,\,\omega_{\theta }^{(2)}$ are the components of velocity and vorticity of the spherical cavity $a_{2}$ without the solid particle $a_{1}$ as shown in Fig. 1. Additionally, the subsequent relations link the coordinate systems $(r_{1} ,\theta_{1} )$ and $(r_{2} ,\theta_{2} )$ together as:

30 $$ r_{1}^{2} = r_{2}^{2} + h^{2} - 2r_{2} h\cos \theta_{2} \,, $$

31 $$ r_{2}^{2} = r_{1}^{2} + h^{2} + 2r_{1} h\cos \theta {}_{1}\,. $$

Hence, the boundary conditions are linear so the principle of superposition can be applied. Thus, the field functions are represented as:

32 $$ \left[ \begin{aligned} u_{\phi } (r_{1} ,\theta_{1} ;r_{2} ,\theta_{2} ) \hfill \\ m_{r\theta } (r_{1} ,\theta_{1} ;r_{2} ,\theta_{2} ) \hfill \\ t_{r\phi } (r_{1} ,\theta_{1} ;r_{2} ,\theta_{2} ) \hfill \\ \end{aligned} \right]\,\,\, = \left[ \begin{aligned} u_{\phi }^{(1)} (r_{1} ,\theta_{1} ) \hfill \\ m_{r\theta }^{(1)} (r_{1} ,\theta_{1} ) \hfill \\ t_{r\phi }^{(1)} (r_{1} ,\theta_{1} ) \hfill \\ \end{aligned} \right] + \left[ \begin{aligned} u_{\phi }^{(2)} (r_{2} ,\theta_{2} ) \hfill \\ m_{r\theta }^{(2)} (r_{2} ,\theta_{2} ) \hfill \\ t_{r\phi }^{(2)} (r_{2} ,\theta_{2} ) \hfill \\ \end{aligned} \right], $$

The next boundary conditions are recommended for the two spherical surfaces:

33 $$ \left. {q_{\phi } } \right|_{{_{{r_{1} = a_{1} }} }} - \frac{1}{{\beta_{1} }}t_{r\phi } = a_{1} \Omega_{1} \sin \theta_{1} ,\,\,\,\,\,\,\,\,\,\left. {m_{r\theta } } \right|_{{_{{r_{1} = a_{1} }} }} = 0, $$

34 $$ \left. {q_{\phi } } \right|_{{_{{r_{2} = a2}} }} + \frac{1}{{\beta_{2} }}t_{r\phi } = a_{2} \Omega_{2} \sin \theta_{2} ,\,\,\,\,\left. {\,\,\,\,\,\,\,\,m_{r\theta } } \right|_{{_{{r_{2} = a_{2} }} }} = 0. $$

From (32) and use (18), (22), (28) the field functions $q_{\phi } ,\,\omega_{r} ,\,\omega_{\theta } ,\,m_{r\theta } ,t_{r\phi }$ are:

35 $$ \begin{aligned} u_{\phi } & = \sum\limits_{n = 1}^{\infty } {\left[ {A_{n} \,\,r_{1}^{ - n - 1} + D_{n} \,\,r_{1}^{{\frac{ - 1}{2}}} K_{{n + \frac{1}{2}}} (\kappa \,r_{1} )} \right]} \,\,P_{n}^{1} \left( {\zeta_{1} } \right) \hfill \\ & \quad  + \sum\limits_{n = 1}^{\infty } {\left[ {B_{n} \,r_{2}^{n} + C_{n} \,\,r_{2}^{{\frac{ - 1}{2}}} \,I_{{n + \frac{1}{2}}} (\kappa \,r_{2} )} \right]} \,\,P_{n}^{1} \left( {\zeta_{2} } \right), \hfill \\ \end{aligned} $$

36 $$ \begin{aligned} \omega_{r} & = \tfrac{1}{2}\sum\limits_{n = 1}^{\infty } {n\left( {n + 1} \right)\left[ {A_{n} \,\,r_{1}^{ - n - 2} + D_{n} \,\,r_{1}^{{\tfrac{ - 3}{2}}} K_{{n + \tfrac{1}{2}}} (\kappa \,r_{1} )} \right]} \,\,P_{n} \left( {\zeta_{1} } \right) \hfill \\ & \quad + \tfrac{1}{2}\sum\limits_{n = 1}^{\infty } {n\left( {n + 1} \right)\left[ {B_{n} \,r_{2}^{n - 1} + C_{n} \,\,r_{2}^{{\tfrac{ - 3}{2}}} I_{{n + \tfrac{1}{2}}} (\kappa \,r_{2} )} \right]} \,\,P_{n} \left( {\zeta_{2} } \right). \hfill \\ \end{aligned} $$

37 $$ \begin{aligned} \omega_{\theta } & = \tfrac{1}{2}\sum\limits_{n = 1}^{\infty } {\left[ {nA_{n} \,\,r_{1}^{ - n - 2} + D_{n} \,\,r_{1}^{{\tfrac{ - 3}{2}}} \left( {nK_{{n + \tfrac{1}{2}}} \left( {\kappa r_{1} } \right) + \kappa r_{1} K_{{n - \tfrac{1}{2}}} \left( {\kappa r_{1} } \right)} \right)} \right]} \,\,P_{n}^{1} \left( {\zeta_{1} } \right) \hfill \\ & \quad + \tfrac{1}{2}\sum\limits_{n = 1}^{\infty } {\left[ { - (n + 1)B_{n} \,r_{2}^{n - 1} + C_{n} \,\,r_{2}^{{\tfrac{ - 3}{2}}} \left( {nI_{{n + \tfrac{1}{2}}} \left( {\kappa r_{2} } \right) - \kappa r_{2} I_{{n - \tfrac{1}{2}}} \left( {\kappa r_{2} } \right)} \right)} \right]} \,\,P_{n}^{1} \left( {\zeta_{2} } \right). \hfill \\ \end{aligned} $$

38 $$ \begin{aligned} m_{r\theta } &= - 2\sum\limits_{n = 1}^{\infty } {\,\,P_{n}^{1} \left( {\zeta_{1} } \right)\left[ {n(n + 2)(\eta + \eta^{\prime})r_{1}^{ - n - 3} A_{n} } \right.} \hfill \\ & \quad \left. { + r_{1}^{{\tfrac{ - 5}{2}}} \left( {\left( {\kappa^{2} r_{1}^{2} \eta + n(n + 2)\left( {\eta + \eta^{\prime}} \right)} \right)K_{{n + \tfrac{1}{2}}} (\kappa r_{1} ) + \kappa r_{1} \left( {\eta + \eta^{\prime}} \right)K_{{n - \tfrac{1}{2}}} (\kappa r_{1} )} \right)D_{n} } \right] \hfill \\ & \quad - 2\sum\limits_{n = 1}^{\infty } {P_{n}^{1} \left( {\zeta_{2} } \right)\,\,\left[ {(n^{2} - 1)(\eta + \eta^{\prime})r_{2}^{n - 2} B_{n} } \right.} \hfill \\ & \quad \left. { + r_{2}^{{\tfrac{ - 5}{2}}} \left( {\left( {\kappa^{2} r_{2}^{2} \eta + n(n + 2)\left( {\eta + \eta^{\prime}} \right)} \right)I_{{n + \tfrac{1}{2}}} (\kappa r_{2} ) - \kappa r_{2} \left( {\eta + \eta^{\prime}} \right)\,I_{{n - \tfrac{1}{2}}} (\kappa r_{2} )} \right)C_{n} } \right]. \hfill \\ \end{aligned} $$

39 $$ \begin{aligned} T_{r\phi } & = \,\sum\limits_{n = 1}^{\infty } {P_{n}^{1} (\zeta_{1} )\left[ {A_{n} r_{r}^{ - n - 4} \left( { - n\,(n + 1)(2\eta - n\eta^{\prime}) - \mu r_{1}^{2} (n + 2) - \alpha_{{1r_{1} }} } \right)} \right.} \hfill \\ & \quad - D_{n} r_{1}^{{\tfrac{ - 7}{2}}} \left( {\kappa r_{1} \left( {\mu r_{1}^{2} - n\left( {\eta + \eta^{\prime}} \right)\left( {n + 1} \right) - (\kappa^{2} r_{1}^{2} + n)\eta - n(n + 1)\eta^{\prime} + \alpha_{{3r_{1} }} } \right)K_{{n - \tfrac{1}{2}}} (\kappa r_{1} )} \right. \hfill \\ & \quad \left. {\left. { + \left( {\mu \kappa r_{1}^{2} (n + 2) - n(\kappa^{2} r_{1}^{2} + 3n)\eta + n(n + 1)(2\eta - n\eta^{\prime}) - \alpha_{{4r_{1} }} } \right)K_{{n + \tfrac{1}{2}}} (\kappa r_{1} )} \right)} \right] \hfill \\ & \quad + \,\sum\limits_{n = 1}^{\infty } {P_{n}^{1} (\zeta_{2} )\left[ {B\,r_{2}^{n - 3} \left( { - n(n + 1)(2\eta + (n + 1)\eta^{\prime}) + \mu r_{2}^{2} (n - 1) + \alpha_{{2r_{2} }} } \right)} \right.} \hfill \\ & \quad + C_{n} \,r_{2}^{{\tfrac{ - 7}{2}}} \left( {\kappa r_{2} \left( {\mu r_{2}^{2} + \left( {\eta + \eta^{\prime}} \right)n\left( {n + 1} \right) - (\kappa^{2} r_{2}^{2} + n)\eta - n(n + 1)\kappa r_{2} \,\eta^{\prime} + \alpha_{{5r_{2} }} } \right)I_{{n - \tfrac{1}{2}}} (\kappa r_{2} )} \right. \hfill \\ & \quad \left. {\,\left. { + \left( { - \mu r_{2}^{2} (n + 2) + n(\kappa^{2} r_{2}^{2} + 3n)\eta - n(n + 1)(2\eta - n\eta^{\prime}) + \alpha_{{6r_{2} }} } \right)I_{{n + \tfrac{1}{2}}} (\kappa r_{2} )} \right)} \right]. \hfill \\ \end{aligned} $$

Accordingly, by applying the boundary conditions (34) and (35) to Eqs. (35), (38), and (39), we get the following system:

40 $$ \begin{aligned}  & \sum\limits_{n = 1}^{\infty } {P_{n}^{1} (\zeta_{1} )\left[ {A_{n} \left\{ {a_{1}^{ - n - 1} - \beta_{1}^{ - 1} a_{1}^{ - n - 4} \left( { - n\,(n + 1)(2\eta - n\eta^{\prime}) - \mu a_{1}^{2} (n + 2) - \alpha_{{1a_{1} }} } \right)} \right\}} \right.} \hfill \\ &  \quad  \quad + D_{n} a_{1}^{{\tfrac{ - 7}{2}}} \left\{ {\,\beta_{1}^{ - 1} \left( {\kappa a_{1} \left( {\mu a_{1}^{2} - n\left( {\eta + \eta^{\prime}} \right)\left( {n + 1} \right) - (\kappa^{2} a_{1}^{2} + n)\eta - n(n + 1)\eta^{\prime} + \alpha_{{3a_{1} }} } \right)K_{{n - \tfrac{1}{2}}} (\kappa a_{1} )} \right.} \right. \hfill \\ &  \quad  \quad \left. {\left. {\left. { + a_{1}^{{\tfrac{ - 1}{2}}} K_{{n + \tfrac{1}{2}}} (\kappa a_{1} ) + \beta_{1}^{ - 1} \left( {\mu \kappa a_{1}^{2} (n + 2) - n(\kappa^{2} a_{1}^{2} + 3n)\eta + n(n + 1)(2\eta - n\eta^{\prime}) - \alpha_{{4a_{1} }} } \right)K_{{n + \tfrac{1}{2}}} (\kappa a_{1} )} \right)} \right\}} \right] \hfill \\ &  \quad  \quad+ \,\sum\limits_{n = 1}^{\infty } {P_{n}^{1} (\zeta_{2} )\left[ {\,B_{n} \left( {r_{2}^{n} + \beta_{1}^{ - 1} \,r_{2}^{n - 3} \left( { - n(n + 1)(2\eta + (n + 1)\eta^{\prime}) + \mu r_{2}^{2} (n - 1) + \alpha_{{2r_{2} }} } \right)} \right)} \right.} \hfill \\ &  \quad  \quad + C_{n} \,r_{2}^{{\tfrac{ - 7}{2}}} \left\{ { - \beta_{1}^{ - 1} \left( {\kappa r_{2} \left( {\mu r_{2}^{2} + \left( {\eta + \eta^{\prime}} \right)n\left( {n + 1} \right) - (\kappa^{2} r_{2}^{2} + n)\eta - n(n + 1)\kappa r_{2} \,\eta^{\prime} + \alpha_{{5r_{2} }} } \right)I_{{n - \tfrac{1}{2}}} (\kappa r_{2} )} \right.} \right. \hfill \\ &  \quad  \quad \left. {\left. {\,\left. {\left. { + \,r_{2}^{{\tfrac{ - 1}{2}}} \,I_{{n + \tfrac{1}{2}}} (\kappa \,r_{2} ) - \beta_{1}^{ - 1} \left( { - \mu r_{2}^{2} (n + 2) + n(\kappa^{2} r_{2}^{2} + 3n)\eta - n(n + 1)(2\eta - n\eta^{\prime}) + \alpha_{{6r_{2} }} } \right)I_{{n + \tfrac{1}{2}}} (\kappa r_{2} )} \right)} \right\}} \right]} \right|_{{r_{1} = a_{1} }} \hfill \\ &  \quad  \quad = a_{1} \Omega_{1} \sqrt {1 - \zeta_{1}^{2} } \,\,\,, \hfill \\ \end{aligned} $$

41 $$ \begin{aligned} & \sum\limits_{n = 1}^{\infty } {P_{n}^{1} (\zeta_{1} )\left[ {A_{n} \left\{ {r_{1}^{ - n - 1} + \beta_{2}^{ - 1} r_{1}^{ - n - 4} \left( { - n\,(n + 1)(2\eta - n\eta^{\prime}) - \mu r_{1}^{2} (n + 2) - \alpha_{{1r_{1} }} } \right)} \right\}} \right.} \hfill \\ & \quad  \quad + D_{n} r_{1}^{{\tfrac{ - 7}{2}}} \left\{ {\, - \beta_{2}^{ - 1} \left( {\kappa r_{1} \left( {\mu r_{1}^{2} - n\left( {\eta + \eta^{\prime}} \right)\left( {n + 1} \right) - (\kappa^{2} r_{1}^{2} + n)\eta - n(n + 1)\eta^{\prime} + \alpha_{{3r_{1} }} } \right)K_{{n - \tfrac{1}{2}}} (\kappa r_{1} )} \right.} \right. \hfill \\ & \quad  \quad \left. {\left. {\left. {\left. { + r_{1}^{{\tfrac{ - 1}{2}}} K_{{n + \tfrac{1}{2}}} (\kappa \,r_{1} ) - \beta_{2}^{ - 1} \left( {\mu \kappa r_{1}^{2} (n + 2) - n(\kappa^{2} r_{1}^{2} + 3n)\eta + n(n + 1)(2\eta - n\eta^{\prime}) - \alpha_{{4r_{1} }} } \right)K_{{n + \tfrac{1}{2}}} (\kappa r_{1} )} \right)} \right\}} \right]} \right|_{{r_{2} = a_{2} }} \hfill \\ & \quad  \quad + \,\sum\limits_{n = 1}^{\infty } {P_{n}^{1} (\zeta_{2} )\left[ {\,B_{n} \left( {a_{2}^{n} - \beta_{2}^{ - 1} a_{2}^{n - 3} \left( { - n(n + 1)(2\eta + (n + 1)\eta^{\prime}) + \mu a_{2}^{2} (n - 1) + \alpha_{{2a_{2} }} } \right)} \right)} \right.} \hfill \\ & \quad  \quad + C_{n} \,a_{2}^{{\tfrac{ - 7}{2}}} \left\{ {\beta_{2}^{ - 1} \left( {\kappa a_{2} \left( {\mu a_{2}^{2} + \left( {\eta + \eta^{\prime}} \right)n\left( {n + 1} \right) - (\kappa^{2} a_{2}^{2} + n)\eta - n(n + 1)\kappa a_{2} \,\eta^{\prime} + \alpha_{{5a_{2} }} } \right)I_{{n - \tfrac{1}{2}}} (\kappa a_{2} )} \right.} \right. \hfill \\ & \quad  \quad \left. {\,\left. {\left. { + \,a_{2}^{{\tfrac{ - 1}{2}}} \,I_{{n + \tfrac{1}{2}}} (\kappa a_{2} ) + \beta_{2}^{ - 1} \left( { - \mu a_{2}^{2} (n + 2) + n(\kappa^{2} a_{2}^{2} + 3n)\eta - n(n + 1)(2\eta - n\eta^{\prime}) + \alpha_{{6a_{2} }} } \right)I_{{n + \tfrac{1}{2}}} (\kappa a_{2} )} \right)} \right\}} \right] \hfill \\ & \quad  \quad= a_{2} \Omega_{2} \sqrt {1 - \zeta_{2}^{2} } \,\,\,, \hfill \\ \end{aligned} $$

42 $$ \begin{aligned} &  \sum\limits_{n = 1}^{\infty } {\left[ {n(n + 2)(\eta + \eta^{\prime})a_{1}^{ - n - 3} A_{n} } \right. + a_{1}^{{\tfrac{ - 5}{2}}} \left\{ {\left( {\ell^{2} a_{1}^{2} \eta + n(n + 2)(\eta + \eta^{\prime})} \right)K_{{n + \tfrac{1}{2}}} (\ell a_{1} )} \right.} \hfill \\ & \quad \quad \left. { + \left. {\ell a_{1} \left( {\eta + \eta^{\prime}} \right)K_{{n - \tfrac{1}{2}}} (\ell a_{1} )} \right\}D_{n} } \right]P_{n}^{1} (\zeta_{1} ) \hfill \\ & \quad \quad + \sum\limits_{n = 1}^{\infty } {\left[ {(n^{2} - 1)(\eta + \eta^{\prime})r_{2}^{n - 2} B_{n} } \right. + r_{2}^{{\tfrac{ - 5}{2}}} \left\{ {\left( {\ell^{2} r_{2}^{2} \eta + n(n + 2)(\eta + \eta^{\prime})} \right)I_{{n + \tfrac{1}{2}}} (\ell r_{2} )} \right.} \hfill \\ & \quad \quad \left. { - \left. {\left. {\ell r_{2} \left( {\eta + \eta^{\prime}} \right)I_{{n - \tfrac{1}{2}}} (\ell r_{2} )} \right\}C_{n} } \right]P_{n}^{1} (\zeta_{2} )} \right|_{{r_{1} = a_{1} }} = 0\,\,, \hfill \\ \end{aligned} $$

43 $$ \begin{aligned} & \sum\limits_{n = 1}^{\infty } {\left[ {n(n + 2)(\eta + \eta^{\prime})r_{1}^{ - n - 3} A_{n} } \right. + r_{1}^{{\tfrac{ - 5}{2}}} \left\{ {\left( {\ell^{2} r_{1}^{2} \eta + n(n + 2)(\eta + \eta^{\prime})} \right)K_{{n + \tfrac{1}{2}}} (\ell r_{1} )} \right.} \hfill \\ & \quad \quad \left. {\left. { + \left. {\ell r_{1} \left( {\eta + \eta^{\prime}} \right)K_{{n - \tfrac{1}{2}}} (\ell r_{1} )} \right\}D_{n} } \right]P_{n}^{1} (\zeta_{1} )} \right|_{{r_{2} = a_{2} }} \hfill \\ & \quad \quad + \sum\limits_{n = 1}^{\infty } {\left[ {(n^{2} - 1)(\eta + \eta^{\prime})a_{2}^{n - 2} B_{n} } \right. + a_{2}^{{\tfrac{ - 5}{2}}} \left\{ {\left( {\ell^{2} a_{2}^{2} \eta + n(n + 2)(\eta + \eta^{\prime})} \right)I_{{n + \tfrac{1}{2}}} (\ell a_{2} )} \right.} \hfill \\ & \quad \quad - \left. {\left. {\ell a_{2} \left( {\eta + \eta^{\prime}} \right)I_{{n - \tfrac{1}{2}}} (\ell a_{2} )} \right\}C_{n} } \right]P_{n}^{1} (\zeta_{2} ) = 0,\, \hfill \\ \end{aligned} $$

where

$$ \begin{aligned} \alpha_{0r} &= (n^{2} \eta - 2\eta^{\prime})\cot^{2} \theta + ((1 - 2n^{2} )\eta + (3 - n^{2} )\eta^{\prime})\csc^{2} \theta \hfill \\ \alpha_{1r} &= n(n + 1)(n + 2)(\eta + \eta^{\prime}) - 3n^{2} \eta + n\alpha_{0r} \hfill \\ \alpha_{2r} & = ((n^{2} - 2)\eta - 2\eta^{\prime})\cot^{2} \theta + ((3 - 2n^{2} )\eta + (3 - n^{2} )\eta^{\prime})\csc^{2} \theta \hfill \\ \alpha_{3r} & = n(n + 1)(n + 2)(\eta + \eta^{\prime}) - n\alpha_{0} \hfill \\ \alpha_{4r} & = (n + 1)\left[ {n(n - 1)(\eta + \eta^{\prime}) + ((n^{2} - n - 2)\eta - 2\eta^{\prime})\cot^{2} \theta - ((2n^{2} - n + 3)\eta + (n^{2} - 3)\eta^{\prime})\csc^{2} \theta } \right] \hfill \\ \alpha_{5r} & = - (n + 2) + \alpha_{2r} ,\,\,\,\,\,\alpha_{6r} = 3n^{2} \eta - \alpha_{1r} \hfill \\ \end{aligned} $$

These constants $A_{n} ,\,B_{n} ,C_{n} ,D_{n}$ obtained by solving 4N simultaneous linear algebraic Eqs. (40)–(43) provided by the infinite series has been truncated to N terms. To satisfy the boundary criteria at a limited number of discrete locations on the generating arcs of the spherical boundaries, the boundary collocation technique will then be used. The desired unknowns $A_{n} ,\,B_{n} ,C_{n} ,D_{n}$ are then determined by numerically solving the resultant system of equations using the Gauss elimination technique. On the semi-circular longitudinal arc of each particle surface from $\theta = 0$ to $\theta = \pi$, the collocation technique (Ganatos et al. 1980) applies the boundary conditions at a finite number of individual points and reduces the infinite series in Eqs. (40)–(43). The coefficients matrix becomes unique if these points are employed, as shown by looking at the system of linear algebraic equations for the unknown constants $A_{n} ,\,B_{n} ,C_{n} ,D_{n}$. The strategy suggested in the literature, such as that used by Ganatos et al. in 1980 to choose the collocation points, is what we apply to avoid this singular matrix and obtain high accuracy: Four fundamental collocation points on each spherical particle are taken at $\theta_{i} = \varepsilon ,\,{\pi /{2 - }}\varepsilon ,{\pi/{2 + }}\varepsilon ,\pi - \varepsilon$ on the half unit circle $0 \le \theta_{i} \le \pi$ at n any meridian plane, where $\varepsilon$ is provided by a minimal number to prevent the singularity at $\theta_{i} = 0,\,{\pi/2},\pi$. The other points are chosen as mirror-image pairs with $\theta_{i} = {\pi/2}$ and are uniformly spaced around the two-quarter circles, omitting those singularities. The linear algebraic equations are solved using the Gaussian elimination approach to uncover the unknown coefficients, and the hydrodynamic drag force is then calculated. It is sufficient to have $N = 45$ collocations for convergence to occur. To figure out the torque that the fluid on the sphere $a_{1}$ experiences as a result of the tangential stress, we must provide the value of the constant , $\,A_{1}$ which is one of an infinite number of unknown constants.

44 $$ T_{z}^{(1)} = 8\pi \mu \,A_{1} . $$

A spherical particle of radius, $a_{1}$ with angular velocity, $\Omega_{1}$ rotating into an unbounded area of an incompressible viscous fluid flow experiences the following torque:

45 $$ T_{\infty }^{(1)} = 8\pi \mu a_{1}^{3} \Omega_{1} . $$

## Numerical results

**Figure 2:**
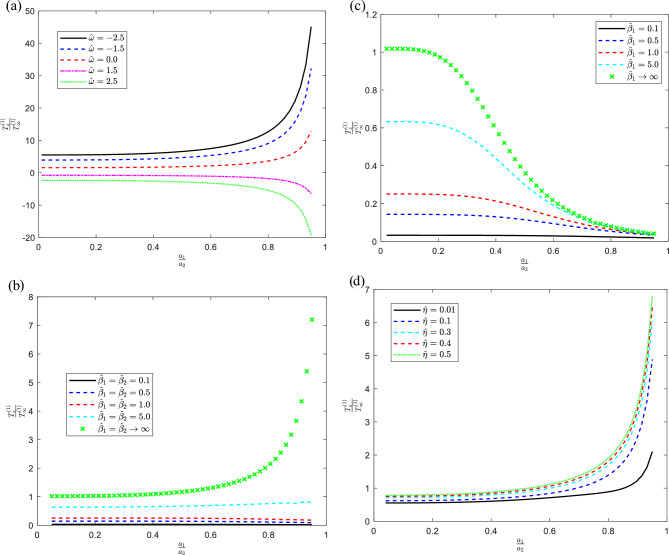
For various values of the indicated parameters, the distribution of torque on the sphere versus the size ratio with (**a**) $\hat{\eta } = 0.5,\,\,\,\hat{\eta }^{\prime} = 0.1,\,\,\hat{\beta }_{1} = \hat{\beta }_{2} \to \infty ,\,\delta = 0.01$, (**b**) $\delta = 0.1,\hat{\eta }^{\prime} = 0,\,\,\hat{\eta } = 0.01,\,\hat{\omega } = 0$, (**c**) $\delta = 0.01,\hat{\beta }_{2} = 0.1,\hat{\eta } = 0.01,\hat{\eta }^{\prime} = 0,\,\hat{\omega } = 0$, (**d**) $\delta = 0.5,\hat{\beta }_{2} = \hat{\beta }_{2} \to \infty ,\hat{\eta }^{\prime} = 0.1,\,\hat{\omega } = 0$.

**Figure 3:**
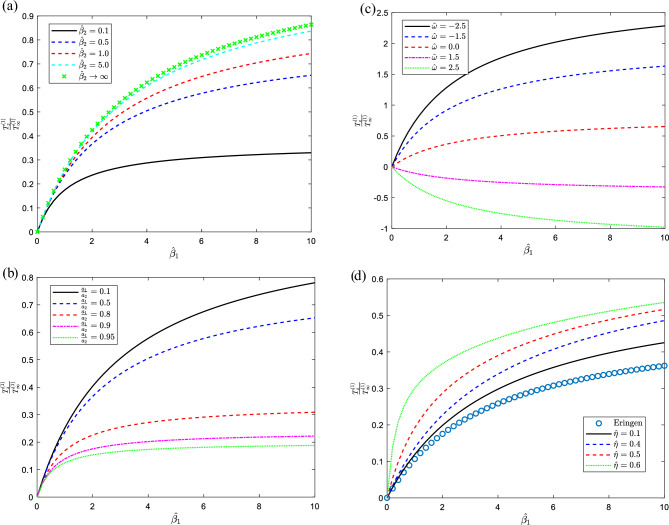
For various values of the indicated parameters, the distribution of torque on the sphere versus the size ratio with (**a**) $\hat{\eta } = 0.01,\,\,\,\hat{\eta }^{\prime} = 0,\,\,{{a_{1} }/{a_{2} = 0.5}},\,\delta = 0.01,\,\hat{\omega } = 0$, (**b**) $\hat{\eta } = 0.01,\,\,\,\hat{\eta }^{\prime} = 0,\,\,\hat{\beta }_{2} = 0.5,\,\delta = 0.01,\,\hat{\omega } = 0$ (**c**) $\hat{\eta } = 0.01,\,\,\,\hat{\eta }^{\prime} = 0,\,\,\hat{\beta }_{2} = 0.5,\,\delta = 0.01,{{a_{1} }/{a_{2} = 0.5}}$, (**d**) $\hat{\omega } = 0.5,\,\,\,\hat{\eta }^{\prime} = 0.5,\,\,\hat{\beta }_{2} = 1,\,\delta = 0.01,{{a_{1} }/{a_{2} = 0.5}}$.

**Figure 4:**
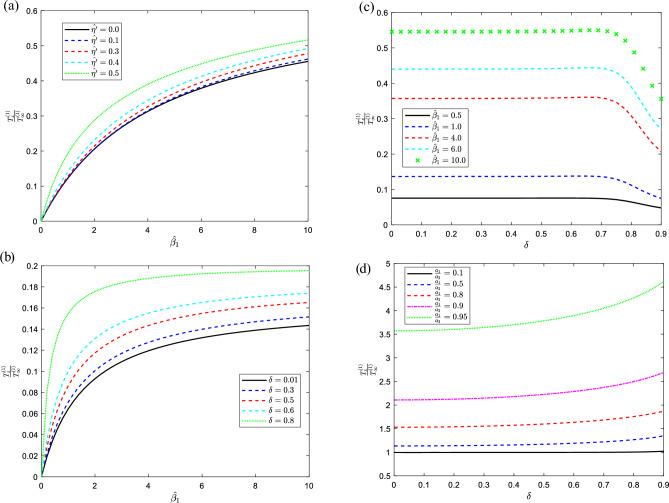
For various values of the indicated parameters, the distribution of torque on the sphere versus the size ratio with (**a**) $\hat{\omega } = 0.5,\,\,\,\hat{\eta } = 0.5,\,\,\hat{\beta }_{2} = 1,\,\delta = 0.01,{{a_{1} }/{a_{2} = 0.5}}$, (**b**) $\hat{\eta }^{\prime} = 0.5,\,\,\hat{\eta } = 0.5,\,\hat{\beta }_{2} = 0.1,\hat{\omega } = 0.5,{{a_{1} }/{a_{2} = 0.5}}$ and versus the separation parameter at (**c**) $\hat{\eta } = 0.8,\,\,\,\hat{\eta }^{\prime} = 0.5,\,\hat{\omega } = 0.5\,,{{a_{1} }/{a_{2} = 0.1}},\,\delta = 0.01$, (**d**) $\hat{\eta } = 0.01,\,\,\,\hat{\eta }^{\prime} = 0.1,\,\hat{\omega } = 0.1\,\hat{\beta }_{1} = \hat{\beta }_{2} \to \infty$.

All results produced using this collocation approach in the present section converge to at least five decimal places. We want to quantitatively represent the normalized torque operating on the inner solid sphere for a variety of parameter values found in the equations governing the phenomenon, the slippage $${{\hat{\beta }_{1} = \beta_{1} }/{\mu a_{1}^{2} ,\,{{\hat{\beta }_{2} = \beta_{2} }/{\mu a_{1}^{2} }}}},$$$$0 \le \,\hat{\beta }_{1} ,\hat{\beta }_{2} \le \infty$$, couple stress of first and second kind the size ratio of the particles $${{a_{1} } /{a_{2} }},\,\,\,0.1 \le {{a_{1} }/{a_{2} }} \le 0.99$$, the separation parameter $$\delta = {h /{(a_{2} - a_{1} )}},$$$$0.001 \le \delta \le 0.9$$, and the angular velocity ratio, $$\hat{\omega } = {{\Omega_{2} }/{\Omega_{1} }},$$$$- 2.5 \le \hat{\omega } \le 2.5$$ parameters. The impact of the relevant parameters within the problem is illustrated graphically in Figs. 2, 3, 4 and numerically in Tables 1, 2 and 3.

**Table 1:** The non-dimensional torque exerted on the internal sphere in this situation when the exterior sphere is stable and the interior sphere is spinning, with zero second couple stress and $\hat{\eta } = 0.01$.

$$T_{z}^{(1)} /T_{\infty }^{(1)}$$							
$\delta$	${{a_{1} }/{a_{2} }}$	$\hat{\beta }_{2} = 1.0$			$\hat{\beta }_{2} \to \infty$ Al-Hanaya et al.^6^		
		$\hat{\beta }_{1} = 0.1$	$\hat{\beta }_{1} = 10.0$	$\hat{\beta }_{1} \to \infty$	$\hat{\beta }_{1} = 0.1$	$\hat{\beta }_{1} = 10.0$	$\hat{\beta }_{1} \to \infty$
0.01	0.1	0.03225	0.78092	1.01889	0.03225	0.78110	1.01920
	0.2	0.03225	0.78245	1.02149	0.03225	0.78540	1.02652
	0.3	0.03225	0.78212	1.02093	0.03227	0.79727	1.04690
	0.4	0.03223	0.77278	1.00507	0.03231	0.82139	1.08889
	0.5	0.03218	0.74389	0.95674	0.03237	0.86439	1.16577
	0.6	0.03206	0.68719	0.86493	0.03247	0.93733	1.30245
	0.7	0.03184	0.60230	0.73436	0.03261	1.06188	1.55598
	0.8	0.03155	0.49769	0.58374	0.03281	1.28904	2.09696
	0.9	0.03094	0.39546	0.44762	0.03313	1.77031	3.78003
	0.99	0.03049	0.31242	0.34469	0.03342	3.04746	34.54674
	0.999	0.03044	0.30529	0.33595	0.03349	3.31620	342.44217
0.25	0.1	0.03224	0.78097	1.01898	0.03225	0.78120	1.01938
	0.2	0.03225	0.78248	1.02156	0.03225	0.78599	1.02760
	0.3	0.03225	0.78167	1.02013	0.03226	0.79881	1.04975
	0.4	0.03224	0.77118	1.00224	0.03228	0.82421	1.09424
	0.5	0.03220	0.74113	0.95201	0.03234	0.86865	1.17418
	0.6	0.03210	0.68398	0.85974	0.03243	0.94303	1.31455
	0.7	0.03190	0.59947	0.73046	0.03257	1.06886	1.57299
	0.8	0.03157	0.49643	0.58227	0.03279	1.29714	2.12226
	0.9	0.03119	0.38748	0.44756	0.03313	1.79807	3.8279
	0.99	0.03049	0.31242	0.34468	0.03342	3.04790	34.99001
	0.999	0.03044	0.30529	0.33595	0.03349	3.31620	346.83475
0.5	0.1	0.03224	0.78117	1.01932	0.03224	0.78168	1.02021
	0.2	0.03225	0.78244	1.02149	0.03223	0.78862	1.03230
	0.3	0.03227	0.77951	1.01631	0.03220	0.80499	1.06114
	0.4	0.03229	0.76516	0.99163	0.03218	0.83473	1.11430
	0.5	0.03230	0.73159	0.93578	0.03220	0.88377	1.20443
	0.6	0.03222	0.67352	0.84320	0.03230	0.96257	1.35690
	0.7	0.03205	0.59191	0.71966	0.03248	1.09241	1.63153
	0.8	0.03165	0.49270	0.57840	0.03274	1.28574	2.20844
	0.9	0.03106	0.39340	0.44729	0.03319	1.80238	3.99041
	0.99	0.03049	0.31241	0.34466	0.03342	3.04924	36.48973
	0.999	0.03044	0.30529	0.33594	0.03349	3.31620	361.69635
0.99	0.1	0.03192	0.75623	0.98597	0.03583	0.80028	1.22229
	0.2	0.03365	0.77073	0.99450	0.03398	0.89441	1.23946
	0.3	0.03251	0.71317	0.91287	0.04197	0.92638	1.32305
	0.4	0.02976	0.73038	0.90166	0.03200	0.95732	1.43159
	0.5	0.03337	0.67266	0.84166	0.03190	1.02844	1.58224
	0.6	0.03220	0.62949	0.77043	0.02945	1.11103	1.80706
	0.7	0.03301	0.56414	0.67892	0.03223	1.23987	2.21790
	0.8	0.03155	0.48052	0.56356	0.03236	1.52707	3.01919
	0.9	0.03132	0.39104	0.44771	0.03291	1.59731	5.47414
	0.99	0.03021	0.31235	0.34457	0.03341	3.05446	50.07121
	0.999	0.03044	0.30528	0.33593	0.03349	3.31618	496.26477

It is found from Tables 1, 2, and Fig. 2a represents the torque for no-slippage at certain values of related parameters for various values of angular velocity ratio, so the torque is a force that makes an item rotate around its axis. It is also known as the moment of force. Physically, speed is defined as the amount of distance traveled in each amount of time, but angular velocity refers to the rate of rotation of the body and the number of revolutions in each amount of time. Thus, the non-dimensional torque is inversely proportional to the angular velocity. Consequently, that the torque decreases with increasing, $\hat{\omega }$ which agrees with the physical concepts. in addition, the torque slowly decreases for the positive values of the angular velocity with the increase of size ratio but for the negative values, it changes its direction to up. Figure 2b displays the torque for various values of slippage parameters for fixed the cavity and the solid sphere rotates with value one. Hence, the torque increases with the increase of both the slippage parameters and the size ratio. This mode is like Motor Mode, the induction motor torque swings in this mode of operation as the slip changes, going from zero to full load torque. From zero to one is the slide. At no load, the value is 0; at rest, it is 1. But for the fluid, the slip parameter varies from zero to infinity, where zero denotes perfect slip, the values in between are the partial slippage, and infinity denotes the no-slip condition, the last is the limiting situation for the work of Amal et al.^6^. The curves show a clear relationship between the torque and the slip. In other words, the amount of torque produced increases with slippage and vice versa. Additionally, Fig. 2c differs from Fig. 2b with the value of the separation parameter, and also the cavity has a partial slip which makes the torque diminish rapidly with the increase of the size ratio, the torque value appears to be minimal when the particle is in a concentric position inside the cavity ($\delta \approx 0$) as expected. We have found that our placement results of the torque in a concentric position are very similar to the analytical solution available in the literature. Moreover. Figure 2d for no-slippage indicated the advancement of torque with the improvement of the first couple stress parameter.

**Table 2:** The non-dimensional torque exerted on the internal sphere in this situation when the exterior sphere is stable and the interior sphere is spinning, with zero second couple stress.

$$T_{z}^{(1)} /T_{\infty }^{(1)}$$							
$\delta$	${{a_{1} }/{a_{2} }}$	$\hat{\beta }_{1} = \hat{\beta }_{2} = 4.0$			$\hat{\beta }_{1} = \hat{\beta }_{2} \to \infty$ Al-Hanaya et al.^6^		
		$\hat{\eta } = 0.01$	$\hat{\eta } = 0.1$	$\hat{\eta } = 0.5$	$\hat{\eta } = 0.01$	$\hat{\eta } = 0.1$	$\hat{\eta } = 0.5$
0.01	0.1	0.57330	0.65116	0.66421	1.01920	1.15055	1.52470
	0.2	0.57524	0.65355	0.66666	1.02652	1.15980	1.54038
	0.3	0.57994	0.65890	0.67213	1.04690	1.18530	1.58215
	0.4	0.58803	0.66702	0.68037	1.08889	1.23713	1.66536
	0.5	0.59962	0.67634	0.68994	1.16577	1.33082	1.81308
	0.6	0.61433	0.68317	0.69826	1.30245	1.49618	2.06809
	0.7	0.63420	0.67755	0.70128	1.55598	1.80220	2.52955
	0.8	0.65267	0.64280	0.67590	2.09696	2.45493	3.49669
	0.9	0.66648	0.61653	0.59771	3.78003	4.48301	6.47011
	0.99	0.66758	0.56537	0.54800	34.54636	56.94579	60.50151
	0.999	0.66921	0.55884	0.54199	342.43909	565.72382	601.04962
0.25	0.1	0.57334	0.65125	0.66431	1.01938	1.15076	1.52505
	0.2	0.57544	0.65408	0.66735	1.02760	1.16105	1.54213
	0.3	0.58037	0.66006	0.67385	1.04975	1.18856	1.58667
	0.4	0.58866	0.66851	0.68537	1.09424	1.24326	1.67390
	0.5	0.60021	0.67762	0.68897	1.17418	1.34056	1.82667
	0.6	0.61478	0.68395	0.69731	1.31455	1.51039	2.08795
	0.7	0.63384	0.66559	0.69843	1.57299	1.82243	2.55800
	0.8	0.66201	0.65454	0.68267	2.12226	2.48523	3.53980
	0.9	0.66384	0.61580	0.59537	3.82797	4.54064	6.55318
	0.99	0.66758	0.56517	0.54865	34.98962	57.67765	61.27888
	0.999	0.66921	0.56884	0.54194	346.83163	572.98175	608.76062
0.5	0.1	0.57355	0.65180	0.66499	1.02021	1.45161	1.52686
	0.2	0.57631	0.65720	0.67172	1.03230	1.47391	1.55118
	0.3	0.58189	0.66552	0.68360	1.06114	1.52388	1.60517
	0.4	0.59175	0.67437	0.60488	1.11430	1.61663	1.70529
	0.5	0.59378	0.68152	0.68926	1.20443	1.77413	1.87480
	0.6	0.62323	0.68501	0.69514	1.35690	2.03751	2.15696
	0.7	0.63511	0.67039	0.69164	1.63153	2.50484	2.65567
	0.8	0.64391	0.66207	0.68389	2.20844	3.47339	3.68662
	0.9	0.66834	0.60611	0.58671	3.99041	6.43495	6.83469
	0.99	0.66757	0.56459	0.54877	36.48932	60.15379	63.90918
	0.999	0.66921	0.55884	0.54199	361.69312	597.53766	634.84937
0.99	0.1	0.56781	0.57258	0.56857	1.21999	1.76665	1.85133
	0.2	0.59375	0.61575	0.61091	1.23978	1.80276	1.90223
	0.3	0.60225	0.64550	0.64169	1.32249	1.88068	2.15113
	0.4	0.61329	0.67245	0.67025	1.42115	2.14798	2.16144
	0.5	0.62772	0.69213	0.68995	1.58843	2.21828	2.48490
	0.6	0.64324	0.69569	0.68939	1.82290	2.73840	2.89574
	0.7	0.65705	0.68240	0.66848	2.21619	3.42169	3.61707
	0.8	0.66846	0.66582	0.64596	3.01909	4.77911	5.06701
	0.9	0.67874	0.65369	0.63172	5.47408	8.86403	9.40760
	0.99	0.66756	0.56445	0.54731	50.05823	496.22067	87.89735
	0.999	0.66921	0.56883	0.54199	496.22067	817.37067	868.80151

Furthermore, Fig. 3a–d illustrate the distribution of torque against the velocity slippage on the solid sphere for certain values of the rest parameters. Figure 3a displays the torque growths with the growth of the slip parameter on the cavity and at the same time increases with the slippage on the solid sphere. On the other hand, in Fig. 3b the torque diminishes with the increase of the size ratio, ${{a_{1} }/{a_{2} }}$ and the torque as mentioned previously increases with the slip parameter. Figure 3c exhibits the torque where the curves diverged when approaching high values of slippage parameters and the torque declines with the increase of the angular velocity ratio as usual. Thus, in Fig. 3d exposits the torque has more significant for the improvement of the first couple stress parameter and agree with the limiting case of viscous fluids. Table 3 shows the exact value was taken when N = 90 for the convergence of the normalized torque with various parameters.

**Table 3:** Convergence of the non-dimensional torque exerted on the internal sphere for different parameters of $\hat{\omega } = 0.5,\,\,\,\hat{\eta } = 0.8,\hat{\eta }^{\prime} = 0.5,\,\,{{a_{1} }/{a_{2} = 0.1}},\delta = 0.5$ with $CPU = 36.55\,\,\,\sec.$

$N$	$\hat{\beta }_{1} = \hat{\beta }_{2} = 0.001$	$\hat{\beta }_{1} = \hat{\beta }_{2} = 1.0$	$\hat{\beta }_{1} = \hat{\beta }_{2} = 4.0$	$\hat{\beta }_{1} = \hat{\beta }_{2} = 6.0$	$\hat{\beta }_{1} = \hat{\beta }_{2} \to \infty$
4	0.00015	0.12565	0.35071	0.43878	0.55054
10	0.00017	0.13649	0.35877	0.44127	0.54684
30	0.00015	0.13715	0.35825	0.44120	0.54689
40	0.00015	0.13720	0.35830	0.44125	0.54692
50	0.00014	0.13722	0.35833	0.44127	0.54693
60	0.00013	0.13724	0.35835	0.44128	0.54694
70	0.00011	0.13726	0.35836	0.44129	0.54695
80	0.00011	0.13727	0.35837	0.44130	0.54695
85	0.00010	0.13727	0.35837	0.44130	–
90	–	–	–	–	–

Finally, the distribution of torque versus velocity slippage on the solid sphere for fixed values of the pertinent parameters is presented in Fig. 4a and b as a result of this investigation, the torque increase with the increase of the second couple stress parameter increases. Additionally, Fig. 4b and c expressed the torque versus the separation parameter where the improvement of torque has been shown in Fig. 3b by increasing the distance from the centers between the solid sphere and the spherical cavity. Therefore, in Fig. 4c the torque is directly proportional to the slippage parameter and this relation affects the torque with the separation distance that the torque inclined with the growth of the separation parameter.

## Conclusion

In this research, we study the interfacial slippage effect and the steady incompressible rotation of a couple stress fluids around a rotating sphere. Therefore, the graphs are used to give a numerical analysis of the torque operating on the inner solid sphere's surface. As a result, raising the couple stress coefficient results in an expected increase in torque. Additionally, it is established that the torque is significantly influenced by the second viscosity parameter. It elevates the torque's worth. In addition, it is shown that the size ratio and separation parameter have more significant on the couple stress fluid flow, especially for small values. Finally, it is determined that the slip parameter has a significant influence in raising the torque value. The motivation for studying flow slip boundary conditions comes from the possible applications in a variety of engineering and applied scientific fields, as well as from a serious grasp of hydrodynamics, which serves as the theoretical basis for the design and construction of nanofluidic devices. Additionally, the development of shale reservoirs depends heavily on a knowledge of slip flow behavior in the nano-porous medium. The future study can be applied to this work in the effect of permeability of porous medium and magnetic field such as in^33^ and^34^. Additionally, the impact of oscillation, the fractional approach and electro-osmotic can be employed in this study such as^35–37^.

## Data Availability

The data that support the findings of this study are available in the article.

## List of symbols

$\vec{u}$: The volume-averaged velocity, ${\text{m/s}}$
$p$: The fluid pressure, ${\text{Pa}}\,\,{\text{or}}\,\,{\text{N/m}}^{2}$
$\mu$: The viscosity of the fluid, ${\text{N}}\;{\text{s/m}}^{2}$
$\eta ,\,\eta^{\prime}$: The 1st and 2nd couple stress viscosity, ${1/{{\text{N}}\;{\text{s}}}}$
$t_{ij} ,\,\,m_{ij}$: The stress tensor and the strain tensor, ${\text{N/m}}^{2}$
$\beta_{1} ,\beta_{2}$: The velocity slippages on the two particles, ${\text{N}}\;{\text{s}}$
